# Tetra-*n*-butyl­ammonium bromide: a redetermination at 150 K addressing the merohedral twinning

**DOI:** 10.1107/S1600536811032612

**Published:** 2011-09-14

**Authors:** Mark R. J. Elsegood

**Affiliations:** a Chemistry Department, Loughborough University, Loughborough, LE11 3TU, England

## Abstract

The redetermined, low temperature (150 K), structure of tetra-*n*-butyl­ammonium bromide, (C_4_H_9_)_4_N^+^·Br^−^, has been found to be merohedrally twinned *via* twin law −1 0 0, 0 − 1 0, 1 0 1. The structure was previously determined, with low precision, no inclusion of H atoms and only the bromide ion refined with anisotropic displacement parameters, by Wang *et al.* (1995 ▶). *Mol. Cryst. Liq. Cryst. Sci. Tech. A*, **264**, 115–129. The redetermined structure has considerably improved precision in all geometrical parameters, has all non-H atoms refined anisotropically, H atoms included, and is isomorphous with the iodide analogue. The structure is otherwise routine, with the shortest cation to anion contacts being between the bromide anion and the C*H* atoms close to the ammonium nitro­gen centre at a distance of *ca*. 2.98–3.11 Å. Each anion makes eight such contacts to four different anions. The *n*-butyl chains are fully extended, adopting an all-*anti* conformation with approximate *S*_4_ point symmetry.

## Related literature

The structure was previously determined by Wang *et al.* (1995 ▶). For the uses of tetra-*n*-alkyl­ammonium salts and the isomorphous structure of tetra-*n*-butyl ammonium iodide, see: Prukała *et al.* (2007 ▶). For a related stucture, see: McMullan & Jeffrey (1959 ▶). For the conformation of *n*-butyl chains, see: Alder *et al.* (1990 ▶). For details of the Cambridge Structural Database, see: Fletcher *et al.* (1996 ▶); Allen (2002 ▶).
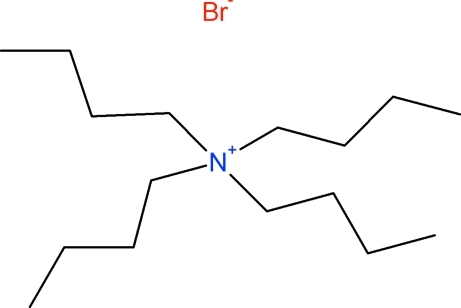


## Experimental

### 

#### Crystal data

C_16_H_36_N^+^·Br^−^
*M* *_r_* = 322.37
Monoclinic, 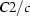

*a* = 13.9773 (9) Å
*b* = 13.8623 (9) Å
*c* = 20.0450 (14) Å
β = 110.383 (10)°
*V* = 3640.7 (4) Å^3^
*Z* = 8
Mo *K*α radiation
μ = 2.25 mm^−1^
*T* = 150 K
0.41 × 0.31 × 0.16 mm

#### Data collection

Bruker APEXII CCD diffractometer
Absorption correction: multi-scan (*SADABS*; Sheldrick, 2008*a* ▶) *T*_min_ = 0.459, *T*_max_ = 0.715
21135 measured reflections
5485 independent reflections
4415 reflections with *I* > 2σ(*I*)
*R*_int_ = 0.029

#### Refinement

*R*[*F* ^2^ > 2σ(*F* ^2^)] = 0.028
*wR*(*F* ^2^) = 0.073
*S* = 1.04
5485 reflections
168 parameters
H-atom parameters constrained
Δρ_max_ = 0.62 e Å^−3^
Δρ_min_ = −0.24 e Å^−3^

### 

Data collection: *APEX2* (Bruker, 2008 ▶); cell refinement: *SAINT* (Bruker, 2008 ▶); data reduction: *SAINT*; program(s) used to solve structure: *SHELXS97* (Sheldrick, 2008*b* ▶); program(s) used to refine structure: *SHELXL97* (Sheldrick, 2008*b* ▶) and *PLATON* (Spek, 2009 ▶); molecular graphics: *SHELXTL* (Sheldrick, 2008*b* ▶); software used to prepare material for publication: *SHELXTL* and local programs.

## Supplementary Material

Crystal structure: contains datablock(s) I, global. DOI: 10.1107/S1600536811032612/rn2089sup1.cif

Structure factors: contains datablock(s) I. DOI: 10.1107/S1600536811032612/rn2089Isup2.hkl

Supplementary material file. DOI: 10.1107/S1600536811032612/rn2089Isup3.cml

Additional supplementary materials:  crystallographic information; 3D view; checkCIF report

## Figures and Tables

**Fig. 1.**
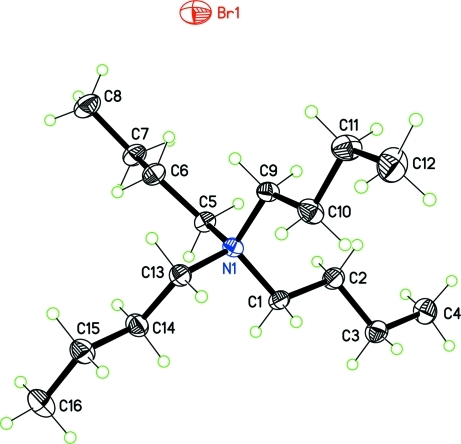
The asymmetric unit in the structure of (I) with displacement ellipsoids drawn at the 50% probability level.

## supplementary crystallographic information

### Comment

While many common reagents have had their crystal structures well determined, some many times, some deliberatly and many by accident, no good quality structure was available for the title compound, tetra-n-butylammonium bromide (I). Compound (I) is used in a number of synthesis applications (see Prukała *et al.*, 2007, and references therein for further details) and as a source of the large tetra-n-butylammonium cation, which is useful in crystallizing large anions. A search of the Cambridge Structural Database (version 5.32 + 3 updates, Fletcher, *et al.*, 1996, Allen, 2002) revealed just one reported structure of this compound with an R1 of 0.098 that had clearly been problematic (Wang *et al.*, 1995). This earlier determination had only the bromide ion refined anisotropically and did not include hydrogen atoms in the model. The authors ruled out dynamic disorder as the cause of the difficulties and concluded that static disorder was the causeof the poor residual.

The crystals of (I) formed readily by vapour diffusion of diethyl ether into an acetonitrile solution. The data collection set-up was trouble free. After data reduction the structure did not solve readily with *SHELXS* (Sheldrick, 2008*a*); only the bromide, the nitrogen and two *n*-butyl chains being evident. When the structure failed to develop, the coordinates from the published structure were used as a starting point (Wang *et al.*, 1995), but the R1 was *ca*. 35% for an isotropic model with all non-H atoms in the model. Twinning was suspected and confirmed by the TWINROTMAT routine in *PLATON* (Spek, 2009). Application of the merohedral twin law -1 0 0, 0 -1 0, 1 0 1, led to a reduction in R1 to *ca*. 5.0% at the same, isotropic, stage of refinement. Anisotropic refinement, and addition of H atoms, led to a good final R1 <3% with no adverse indicators. The ratio of major to minor twin components is 60.69: 39.31 (7)%

The structure is isomorphous with that of the iodide analogue described in detail recently (Prukała *et al.*, 2007). The *n*-butyl chains are fully extended adopting an all-anti conformation with approximate S_4_ point symmetry (Alder *et al.*, 1990). The bromide anion resides in a pocket between four cations, making four pairs of weak C—H···Br contacts in the range 2.98–3.11 Å to methylene hydrogens located one or two carbon atoms from the nitrogen cationic centre. The structures of the chloride and fluoride analogues have not been determined to date, although the unit cell of the hydrate of the chloride has been reported (McMullan & Jeffrey, 1959).

### Experimental

The title compound (I) was used as received and crystallized from an acetonitrile solution *via* vapour diffusion with diethylether to give colourless blocks.

### Refinement

H atoms were included in a riding model with constrained bond lengths: for CH_2_ = 0.99 and CH_3_ = 0.98 Å with *U*_iso_(H) = 1.2 *U*eq(*C*H_2_) and =1.5*U*eq(*C*H_3_).

### Crystal data

**Table:**

C_16_H_36_N^+^·Br^−^	*F*(000) = 1392
*M**_r_* = 322.37	*D*_x_ = 1.176 Mg m^−^^3^
Monoclinic, *C*2/*c*	Mo *K*α radiation, λ = 0.71073 Å
Hall symbol: -C 2yc	Cell parameters from 7468 reflections
*a* = 13.9773 (9) Å	θ = 2.6–30.1°
*b* = 13.8623 (9) Å	µ = 2.25 mm^−^^1^
*c* = 20.0450 (14) Å	*T* = 150 K
β = 110.383 (10)°	Block, colourless
*V* = 3640.7 (4) Å^3^	0.41 × 0.31 × 0.16 mm
*Z* = 8	

### Data collection

**Table:**

Bruker APEXII CCD diffractometer	5485 independent reflections
Radiation source: fine-focus sealed tube	4415 reflections with *I* > 2σ(*I*)
graphite	*R*_int_ = 0.029
ω rotation with narrow frames scans	θ_max_ = 30.5°, θ_min_ = 1.1°
Absorption correction: multi-scan (*SADABS*; Sheldrick, 2008*a*)	*h* = −19→19
*T*_min_ = 0.459, *T*_max_ = 0.715	*k* = −18→19
21135 measured reflections	*l* = −28→28

### Refinement

**Table:**

Refinement on *F*^2^	Primary atom site location: structure-invariant direct methods
Least-squares matrix: full	Secondary atom site location: difference Fourier map
*R*[*F*^2^ > 2σ(*F*^2^)] = 0.028	Hydrogen site location: inferred from neighbouring sites
*wR*(*F*^2^) = 0.073	H-atom parameters constrained
*S* = 1.04	*w* = 1/[σ^2^(*F*_o_^2^) + (0.0379*P*)^2^ + 0.7322*P*] where *P* = (*F*_o_^2^ + 2*F*_c_^2^)/3
5485 reflections	(Δ/σ)_max_ = 0.001
168 parameters	Δρ_max_ = 0.62 e Å^−^^3^
0 restraints	Δρ_min_ = −0.24 e Å^−^^3^

### Special details

**Table:**

Geometry. All e.s.d.'s (except the e.s.d. in the dihedral angle between two l.s. planes) are estimated using the full covariance matrix. The cell e.s.d.'s are taken into account individually in the estimation of e.s.d.'s in distances, angles and torsion angles; correlations between e.s.d.'s in cell parameters are only used when they are defined by crystal symmetry. An approximate (isotropic) treatment of cell e.s.d.'s is used for estimating e.s.d.'s involving l.s. planes.
Refinement. Refinement of *F*^2^ against ALL reflections. The weighted *R*-factor *wR* and goodness of fit *S* are based on *F*^2^, conventional *R*-factors *R* are based on *F*, with *F* set to zero for negative *F*^2^. The threshold expression of *F*^2^ > σ(*F*^2^) is used only for calculating *R*-factors(gt) *etc*. and is not relevant to the choice of reflections for refinement. *R*-factors based on *F*^2^ are statistically about twice as large as those based on *F*, and *R*- factors based on ALL data will be even larger.

### Fractional atomic coordinates and isotropic or equivalent isotropic displacement parameters (Å^2^)

**Table:**

	*x*	*y*	*z*	*U*_iso_*/*U*_eq_	
Br1	0.737682 (14)	0.00074 (2)	0.475441 (8)	0.03037 (6)	
N1	0.49621 (18)	0.25167 (8)	0.49516 (13)	0.0172 (2)	
C1	0.44659 (14)	0.30519 (13)	0.54096 (10)	0.0195 (4)	
H1A	0.4927	0.3579	0.5664	0.023*	
H1B	0.3826	0.3352	0.5093	0.023*	
C2	0.4221 (2)	0.24346 (12)	0.59514 (15)	0.0252 (6)	
H2A	0.4848	0.2105	0.6258	0.030*	
H2B	0.3718	0.1934	0.5702	0.030*	
C3	0.37887 (16)	0.30443 (14)	0.64107 (11)	0.0253 (4)	
H3A	0.3187	0.3406	0.6101	0.030*	
H3B	0.4309	0.3519	0.6682	0.030*	
C4	0.3476 (3)	0.24244 (16)	0.69282 (16)	0.0302 (6)	
H4A	0.2984	0.1936	0.6663	0.045*	
H4B	0.3163	0.2833	0.7194	0.045*	
H4C	0.4081	0.2106	0.7261	0.045*	
C5	0.42140 (14)	0.17734 (13)	0.45040 (10)	0.0191 (4)	
H5A	0.4085	0.1292	0.4827	0.023*	
H5B	0.3559	0.2101	0.4249	0.023*	
C6	0.45519 (19)	0.12433 (12)	0.39615 (10)	0.0230 (4)	
H6A	0.5208	0.0911	0.4206	0.028*	
H6B	0.4659	0.1712	0.3621	0.028*	
C7	0.37484 (16)	0.05075 (14)	0.35601 (11)	0.0259 (4)	
H7A	0.3613	0.0065	0.3905	0.031*	
H7B	0.3104	0.0847	0.3296	0.031*	
C8	0.4089 (3)	−0.00752 (19)	0.30414 (11)	0.0347 (5)	
H8A	0.4195	0.0358	0.2687	0.052*	
H8B	0.3563	−0.0551	0.2801	0.052*	
H8C	0.4729	−0.0409	0.3301	0.052*	
C9	0.59273 (14)	0.19965 (13)	0.54137 (10)	0.0202 (4)	
H9A	0.5729	0.1462	0.5666	0.024*	
H9B	0.6262	0.1708	0.5099	0.024*	
C10	0.6700 (2)	0.26295 (15)	0.59616 (14)	0.0253 (5)	
H10A	0.6917	0.3163	0.5718	0.030*	
H10B	0.6382	0.2914	0.6287	0.030*	
C11	0.76246 (15)	0.20341 (15)	0.63871 (12)	0.0291 (4)	
H11A	0.7914	0.1715	0.6058	0.035*	
H11B	0.7412	0.1526	0.6653	0.035*	
C12	0.8439 (3)	0.26686 (18)	0.69083 (16)	0.0365 (6)	
H12A	0.8637	0.3181	0.6646	0.055*	
H12B	0.9037	0.2275	0.7163	0.055*	
H12C	0.8165	0.2957	0.7251	0.055*	
C13	0.52549 (14)	0.32450 (13)	0.44859 (10)	0.0200 (4)	
H13A	0.5701	0.3741	0.4798	0.024*	
H13B	0.5659	0.2908	0.4238	0.024*	
C14	0.43636 (18)	0.37509 (13)	0.39317 (10)	0.0241 (4)	
H14A	0.3960	0.4108	0.4171	0.029*	
H14B	0.3912	0.3267	0.3611	0.029*	
C15	0.47602 (16)	0.44495 (14)	0.35002 (11)	0.0263 (4)	
H15A	0.5139	0.4086	0.3247	0.032*	
H15B	0.5238	0.4912	0.3826	0.032*	
C16	0.3885 (3)	0.5001 (2)	0.29610 (10)	0.0343 (5)	
H16A	0.3445	0.4549	0.2613	0.051*	
H16B	0.4162	0.5477	0.2716	0.051*	
H16C	0.3486	0.5332	0.3209	0.051*	

### Atomic displacement parameters (Å^2^)

**Table:**

	*U*^11^	*U*^22^	*U*^33^	*U*^12^	*U*^13^	*U*^23^
Br1	0.02772 (9)	0.02330 (8)	0.04278 (10)	0.00074 (10)	0.01565 (8)	0.0010 (2)
N1	0.0158 (9)	0.0157 (5)	0.0192 (6)	−0.0003 (5)	0.0048 (12)	0.0008 (7)
C1	0.0217 (9)	0.0173 (8)	0.0213 (9)	0.0011 (7)	0.0099 (8)	−0.0019 (7)
C2	0.0345 (15)	0.0197 (10)	0.0255 (12)	−0.0008 (7)	0.0157 (11)	0.0002 (7)
C3	0.0274 (10)	0.0261 (9)	0.0271 (10)	0.0023 (8)	0.0154 (8)	0.0008 (8)
C4	0.0316 (15)	0.0361 (12)	0.0308 (12)	0.0011 (9)	0.0208 (14)	0.0059 (10)
C5	0.0177 (9)	0.0181 (8)	0.0221 (10)	−0.0031 (6)	0.0076 (7)	−0.0022 (7)
C6	0.0215 (10)	0.0229 (8)	0.0251 (9)	−0.0023 (8)	0.0088 (9)	−0.0047 (6)
C7	0.0289 (10)	0.0219 (9)	0.0284 (10)	−0.0029 (8)	0.0117 (8)	−0.0061 (8)
C8	0.0435 (15)	0.0270 (10)	0.0368 (9)	−0.0008 (11)	0.0182 (12)	−0.0112 (11)
C9	0.0189 (9)	0.0189 (8)	0.0223 (9)	0.0029 (7)	0.0067 (7)	0.0004 (7)
C10	0.0218 (12)	0.0236 (9)	0.0254 (12)	−0.0009 (8)	0.0017 (10)	0.0011 (8)
C11	0.0193 (9)	0.0299 (10)	0.0341 (11)	0.0015 (8)	0.0042 (8)	−0.0038 (9)
C12	0.0218 (13)	0.0513 (16)	0.0310 (12)	−0.0006 (13)	0.0026 (11)	−0.0076 (12)
C13	0.0227 (10)	0.0169 (8)	0.0227 (10)	−0.0026 (7)	0.0108 (8)	0.0013 (7)
C14	0.0247 (11)	0.0243 (8)	0.0252 (9)	0.0029 (8)	0.0109 (9)	0.0057 (7)
C15	0.0291 (10)	0.0222 (9)	0.0276 (11)	−0.0012 (8)	0.0100 (8)	0.0037 (8)
C16	0.0371 (15)	0.0302 (8)	0.0319 (8)	0.0019 (11)	0.0073 (9)	0.0101 (14)

### Geometric parameters (Å, °)

**Table:**

N1—C5	1.519 (3)	C8—H8B	0.9800
N1—C1	1.522 (3)	C8—H8C	0.9800
N1—C13	1.524 (3)	C9—C10	1.522 (3)
N1—C9	1.526 (3)	C9—H9A	0.9900
C1—C2	1.513 (3)	C9—H9B	0.9900
C1—H1A	0.9900	C10—C11	1.520 (3)
C1—H1B	0.9900	C10—H10A	0.9900
C2—C3	1.521 (3)	C10—H10B	0.9900
C2—H2A	0.9900	C11—C12	1.527 (4)
C2—H2B	0.9900	C11—H11A	0.9900
C3—C4	1.523 (4)	C11—H11B	0.9900
C3—H3A	0.9900	C12—H12A	0.9800
C3—H3B	0.9900	C12—H12B	0.9800
C4—H4A	0.9800	C12—H12C	0.9800
C4—H4B	0.9800	C13—C14	1.521 (3)
C4—H4C	0.9800	C13—H13A	0.9900
C5—C6	1.518 (3)	C13—H13B	0.9900
C5—H5A	0.9900	C14—C15	1.526 (3)
C5—H5B	0.9900	C14—H14A	0.9900
C6—C7	1.523 (3)	C14—H14B	0.9900
C6—H6A	0.9900	C15—C16	1.526 (3)
C6—H6B	0.9900	C15—H15A	0.9900
C7—C8	1.518 (3)	C15—H15B	0.9900
C7—H7A	0.9900	C16—H16A	0.9800
C7—H7B	0.9900	C16—H16B	0.9800
C8—H8A	0.9800	C16—H16C	0.9800
			
C5—N1—C1	108.81 (17)	C7—C8—H8C	109.5
C5—N1—C13	111.35 (18)	H8A—C8—H8C	109.5
C1—N1—C13	108.81 (12)	H8B—C8—H8C	109.5
C5—N1—C9	108.62 (12)	C10—C9—N1	114.89 (16)
C1—N1—C9	110.88 (17)	C10—C9—H9A	108.5
C13—N1—C9	108.39 (18)	N1—C9—H9A	108.5
C2—C1—N1	114.96 (15)	C10—C9—H9B	108.5
C2—C1—H1A	108.5	N1—C9—H9B	108.5
N1—C1—H1A	108.5	H9A—C9—H9B	107.5
C2—C1—H1B	108.5	C11—C10—C9	110.05 (17)
N1—C1—H1B	108.5	C11—C10—H10A	109.7
H1A—C1—H1B	107.5	C9—C10—H10A	109.7
C1—C2—C3	110.93 (15)	C11—C10—H10B	109.7
C1—C2—H2A	109.5	C9—C10—H10B	109.7
C3—C2—H2A	109.5	H10A—C10—H10B	108.2
C1—C2—H2B	109.5	C10—C11—C12	110.9 (2)
C3—C2—H2B	109.5	C10—C11—H11A	109.5
H2A—C2—H2B	108.0	C12—C11—H11A	109.5
C2—C3—C4	111.53 (18)	C10—C11—H11B	109.5
C2—C3—H3A	109.3	C12—C11—H11B	109.5
C4—C3—H3A	109.3	H11A—C11—H11B	108.1
C2—C3—H3B	109.3	C11—C12—H12A	109.5
C4—C3—H3B	109.3	C11—C12—H12B	109.5
H3A—C3—H3B	108.0	H12A—C12—H12B	109.5
C3—C4—H4A	109.5	C11—C12—H12C	109.5
C3—C4—H4B	109.5	H12A—C12—H12C	109.5
H4A—C4—H4B	109.5	H12B—C12—H12C	109.5
C3—C4—H4C	109.5	C14—C13—N1	115.22 (17)
H4A—C4—H4C	109.5	C14—C13—H13A	108.5
H4B—C4—H4C	109.5	N1—C13—H13A	108.5
C6—C5—N1	115.45 (17)	C14—C13—H13B	108.5
C6—C5—H5A	108.4	N1—C13—H13B	108.5
N1—C5—H5A	108.4	H13A—C13—H13B	107.5
C6—C5—H5B	108.4	C13—C14—C15	109.86 (18)
N1—C5—H5B	108.4	C13—C14—H14A	109.7
H5A—C5—H5B	107.5	C15—C14—H14A	109.7
C5—C6—C7	110.28 (19)	C13—C14—H14B	109.7
C5—C6—H6A	109.6	C15—C14—H14B	109.7
C7—C6—H6A	109.6	H14A—C14—H14B	108.2
C5—C6—H6B	109.6	C14—C15—C16	111.1 (2)
C7—C6—H6B	109.6	C14—C15—H15A	109.4
H6A—C6—H6B	108.1	C16—C15—H15A	109.4
C8—C7—C6	111.6 (2)	C14—C15—H15B	109.4
C8—C7—H7A	109.3	C16—C15—H15B	109.4
C6—C7—H7A	109.3	H15A—C15—H15B	108.0
C8—C7—H7B	109.3	C15—C16—H16A	109.5
C6—C7—H7B	109.3	C15—C16—H16B	109.5
H7A—C7—H7B	108.0	H16A—C16—H16B	109.5
C7—C8—H8A	109.5	C15—C16—H16C	109.5
C7—C8—H8B	109.5	H16A—C16—H16C	109.5
H8A—C8—H8B	109.5	H16B—C16—H16C	109.5
			
C5—N1—C1—C2	63.9 (2)	C5—N1—C9—C10	−172.7 (2)
C13—N1—C1—C2	−174.6 (2)	C1—N1—C9—C10	−53.2 (2)
C9—N1—C1—C2	−55.5 (2)	C13—N1—C9—C10	66.2 (2)
N1—C1—C2—C3	176.5 (2)	N1—C9—C10—C11	−179.95 (19)
C1—C2—C3—C4	176.5 (2)	C9—C10—C11—C12	176.4 (2)
C1—N1—C5—C6	174.16 (17)	C5—N1—C13—C14	54.1 (2)
C13—N1—C5—C6	54.2 (2)	C1—N1—C13—C14	−65.8 (2)
C9—N1—C5—C6	−65.0 (2)	C9—N1—C13—C14	173.53 (16)
N1—C5—C6—C7	178.84 (17)	N1—C13—C14—C15	−179.49 (17)
C5—C6—C7—C8	−176.82 (18)	C13—C14—C15—C16	−177.50 (18)
